# 6,6′-Dihydroxythiobinupharidine (DTBN) Purified from *Nuphar lutea* Leaves Is an Inhibitor of Protein Kinase C Catalytic Activity

**DOI:** 10.3390/molecules26092785

**Published:** 2021-05-08

**Authors:** Kamran Waidha, Nikhil Ponnoor Anto, Divya Ram Jayaram, Avi Golan-Goldhirsh, Saravanakumar Rajendran, Etta Livneh, Jacob Gopas

**Affiliations:** 1 Defence Institute of High Altitude Research (DIHAR), Defence Research and Development Organisation (DRDO) Leh, Ladakh UT-194101, India; kamranwaidha1@gmail.com; 2 The Shraga Segal Department of Microbiology, Immunology and Genetics Faculty of Health Sciences, Ben-Gurion University of the Negev, Beer Sheva 8400501, Israel; antop@post.bgu.ac.il (N.P.A.); jayaram@post.bgu.ac.il (D.R.J.); 3 The Jacob Blaustein Institutes for Desert Research (BIDR), Sede Boqer Campus, French Associates Institute for Agriculture and Biotechnology of Drylands, Ben-Gurion University of the Negev, Beer Sheva 8499000, Israel; 4 Chemistry Division, Vellore Institute of Technology Chennai Campus, School of Advanced Sciences, Chennai 600127, India; 5 Department of Oncology, Soroka University Medical Center, Beer Sheva 8400501, Israel

**Keywords:** protein kinase C (PKC), *Nuphar lutea*, 6,6′-dihydroxythiobinupharidine (DTBN), kinase inhibitor, Ramachandran plot, homology docking modeling

## Abstract

Water lily (*Nuphar)* bioactive extracts have been widely used in traditional medicine owing to their multiple applications against human ailments. Phyto-active *Nuphar* extracts and their purified and synthetic derivatives have attracted the attention of ethnobotanists and biochemists. Here, we report that 6,6′-dihydroxythiobinupharidine (DTBN), purified from extracts of *Nuphar lutea* (L.) Sm. leaves, is an effective inhibitor of the kinase activity of members of the protein kinase C (PKC) family using in vitro and in silico approaches. We demonstrate that members of the conventional subfamily of PKCs, PKCα and PKCγ, were more sensitive to DTBN inhibition as compared to novel or atypical PKCs. Molecular docking analysis demonstrated the interaction of DTBN, with the kinase domain of PKCs depicting the best affinity towards conventional PKCs, in accordance with our in vitro kinase activity data. The current study reveals novel targets for DTBN activity, functioning as an inhibitor for PKCs kinase activity. Thus, this and other data indicate that DTBN modulates key cellular signal transduction pathways relevant to disease biology, including cancer.

## 1. Introduction

Natural products of *Nuphar lutea* (L.) Sm. (Nymphaeaceae) have been widely used for treating inflammatory conditions in ethnic medicine [1,2]. A systematic review of early studies revealed that the full therapeutic potential of *Nuphar* products is still largely unexplored by modern research [3]. Nevertheless, recent reports from various laboratories, including ours, on the medicinal properties of semi-purified *Nuphar* extracts (NUP) have indicated potential applications. We have previously published that a semi-purified leaf extract from yellow water lily, *Nuphar lutea* (L.) Sm. (NUP), is enriched in sesquiterpene thioalkaloids, such as nupharidine and bi-nupharidine isomers [4]. A variety of therapeutic applications have been described: anti-inflammatory [2], antibacterial [5,6], antiviral [1], against pathogenic fungi [7], against leishmania, and trypanosome parasites, [8,9,10], and against cancer. We previously examined the effect of NUP extracts in B16 melanoma, experimental murine lung metastasis model, and in a variety of cell lines, demonstrating its ability to affect ERK and NF-κB pathways. We showed that combined NUP and cisplatin treatment was synergistic and reduced the lung metastatic load. In addition, NUP treatment inhibited TNFα-induced IκBα degradation and NF-κB nuclear translocation. We also observed that NUP induced ERK activation. Furthermore, ERK inhibition prevented NF-κB inactivation by NUP [5,11,12]. We have recently published that 6,6′-dihydroxythiobinupharidine (DTBN), purified from NUP, very efficiently and covalently inhibited human type II topoisomerase [13]. Lastly, we recently published that the purified molecule primes neutrophils against bacteria present in gum inflammation, enhances phagocytosis, ROS production, and NET formation [14]. We hypothesize that the mechanism of action of nupharidines goes via the electrophilic thiaspirane warhead of nupharidine(s), which targets a nucleophilic cysteine at the active site of cysteine proteases, and potentially other enzymes. This mechanism may explain the pleiotropic effect of this family of compounds [15]. Being pleiotropic and not limited to a single molecular target may be advantageous.

The protein kinase C (PKC) family comprised a group of serine/threonine kinases which transduce a multitude of signals that control diverse cellular processes, such as proliferation, migration, invasion, differentiation, apoptosis, transcription, and translation [16,17]. Aberrant PKC activity or localization has been linked to numerous diseases, including cancer [18], pain [19], and neurodegenerative diseases [20]. PKC comprises nine genes that share a similar architecture, with an N-terminal regulatory moiety and a C-terminal kinase domain [21,22]. PKC members are classified into three major structurally and functionally distinct subfamilies, including conventional or classical PKC (cPKCs: α, β, and γ), novel PKC (nPKCs: δ, ε, η, θ), and atypical PKC (aPKCs: ι/*λ* and ζ) isoforms [21,22,23].

Numerous observations from basic studies and clinical trials have implicated PKC in the pathophysiology of diverse types of diseases, including cancer [24,25]. PKCs are involved in different signaling pathways, including ERK and NF-kB, suggesting that PKCs could be a target for NUP activity [26].

Our results show that DTBN inhibits PKC isoforms, preferentially conventional PKCs. In silico molecular docking analysis of DTNB to these enzymes confirmed our results, and provides a structural basis for the interaction of DTNB with PKCs.

## 2. Results

### 2.1. Protein Kinase C (PKC) Inhibition Assay

**Figure 1:**
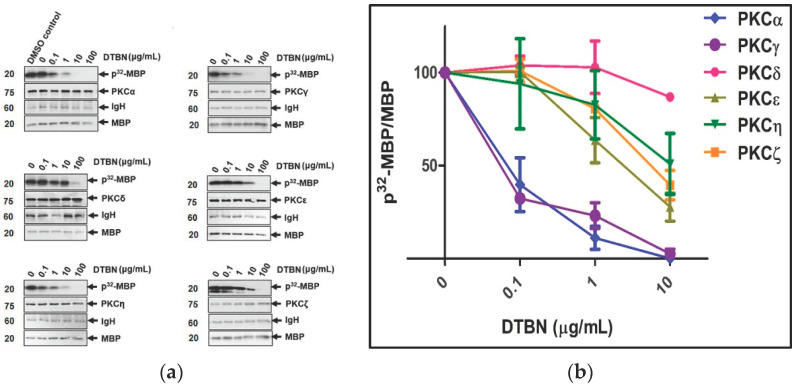
DTBN downregulates PKC catalytic activity in a concentration-dependent manner. In vitro kinase assays of indicated PKC isoforms were performed, as described in Materials and Methods, utilizing MBP as a substrate. Before the initiation of the assay, DTBN was added to the samples at several concentrations. Following the termination of the assay, sample proteins were then resolved by SDS-PAGE and developed by autoradiography (**a**). p^32^-MBP and MBP protein band signals were quantified using Image Lab software, and the relative amounts of p^32^-MBP/MBP were presented in a line graph (**b**). Immunoblotting against HA and MBP to detect overexpressed PKC expression, and MBP, respectively, monitor equal loading of samples. Data are representative of two independent experiments. Molecular weight markers (in kDa) are indicated on the left, and arrows mark the positions of the indicated protein bands. IgH, Ig heavy chain.

To test the effect of DTBN on the activity of PKC isoforms, in vitro kinase assays were performed in a dose–response experiment, and results are presented in Figure 1. At these concentrations, the kinase activities of PKCs were inhibited by DTBN in a dose-dependent manner, having the most profound effect on conventional PKCα and PKCγ as compared to other PKCs tested (Figure 1 and Table 1). To calculate the median inhibitory concentration (IC_50_), the relative amounts of p^32^-MBP/MBP were plotted at several DTBN concentrations: IC_50_ = 0.174 μM (PKCα), 0.168 μM (PKCγ), 14.23 μM (PKCε), 18.6 μM (PKCζ), and >19 μM (PKCη and PKCδ). Immunoblotting was performed with antibodies against HA-tagged different PKCs and against Myelin Basic Protein (MBP) as a loading control.

### 2.2. Molecular Docking of DTBN to PKC Isoforms

Using molecular docking of DTBN to individual PKCs, we demonstrate binding of DTBN to the ATP binding pocket of PKCs, with PKCα exhibiting the deepest insertion into the ATP binding pocket, followed by PKCγ. Post mm-GBSA refinement binding energy (Table 2) strongly correlated with the in vitro results. DTBN has the highest binding energy to conventional PKCs (α and γ) and lowest to PKCδ and ζ. The difference in binding energy may be due to the interaction of DTBN with amino acid residues at the active site and the extent to which the DTBN fits into the ATP binding pocket of PKC isoforms.

## 3. Discussion

The inclusion of DTBN inhibited the ability of PKCs to phosphorylate their substrate MBP, myelin basic protein, in a dose-dependent manner (Figure 1). The inhibitory activity of DTBN was most effective against members of the conventional families PKCα and PKCγ, with PKCα and PKCγ almost equally affected by DTBN, especially at a low drug concentration (IC_50_ = 0.174 and 0.168 μM, Table 1). The novel PKC isozyme, PKCε, was also inhibited by DTBN (IC_50_ = 14.23 μM, Table 1). Interestingly, PKCδ and PKCη, of the novel subclass, were the least affected upon DTBN inclusion in the kinase assay system (IC_50_ >19 μM each, Table 1). Similar observations were also noted on PKCζ (IC_50_ = 18.6 μM, Table 1), a member from the atypical PKC family. Taken together, our results demonstrate DTBN as an inhibitor of PKC catalytic activity, with conventional PKCs being the most affected, especially PKCα and PKCγ.

As mentioned in the introduction, we previously reported the anti-inflammatory, anticancer, and antibacterial properties of *Nuphar* semipurified extracts (NUP) and purified DTBN. Our studies provided evidence for the phytoactive ability of NUP in modulating various cell signaling cascades. For instance, it enhanced ERK activation, which resulted in a decreased activity of TNFα-induced NF-κB. DTBN also was shown to covalently inhibit human type II topoisomerase activity, an observation that highlighted the possibility of DTBN behaving as a potent inhibitor of human enzymes by targeting their active sites [13]. Here, we demonstrate that DTBN is a PKC inhibitor. Considering the inhibitory effect of DTBN on NF-κB, we propose that PKCs, and in particular PKCα and PKCγ, mediate this DTBN effect on NF-κB, and perhaps other signaling cascades in which DTBN was shown to be involved. Indeed, numerous reports linked PKCs to NF-κB signaling in various cancers [27,28,29,30]. For example, PKCα was shown to activate NF-κB, while PKCδ downregulated NF-κB activation [31]. Thus, PKCs could be a target for NUP activity. To understand the varied inhibitory activity of DTBN against PKC isoforms, molecular docking was conducted.

**Figure 2:**
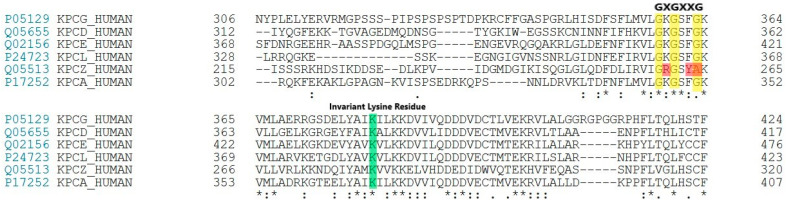
ATP binding kinase domain site alignment and invariant lysine residue of PKC isoforms. Red: Denotes variation in the ATP binding site residue of PKCζ (GRGSYA: Gly264 into Ala264, Phe263 into Tyr263, and Lys260 into Arg260). Yellow: Conserved Glycine Rich region of ATP Binding site across PKC isoforms, Green: Invariant Lysine Residue across PKC isoforms which structures the enzyme for phosphoryl-transfer. * Denotes position which has fully conserved amino acid residues across multiple sequences. “:” Denotes conservation between groups of strong similar properties. “.” Denotes conservation between groups of weakly similar properties.

To understand the inhibitory action and molecular interactions of DTBN with PKC isoforms, DTBN was docked to the ATP binding site of PKC isoforms. PKC catalytic domain consists of an amino (–NH_2_) terminal lobe, which is made up of β-sheets and contains the ATP binding domain, which is rich in glycine and has a consensus sequence of GXGXXG and an invariant lysine. The carboxylic (–COOH) end domain mostly comprises α-helical structures and contains an activation loop. Multiple sequence alignment and ATP binding site of all PKC isoforms (α, δ, η, ε and γ) are highly conserved (GKGSFG), except for PKCζ (Figure 2); there is variation in the ATP binding site sequence (GRGSYA: Gly264 into Ala264, Phe263 into Tyr263, and Lys260 into Arg260).

**Figure 3:**
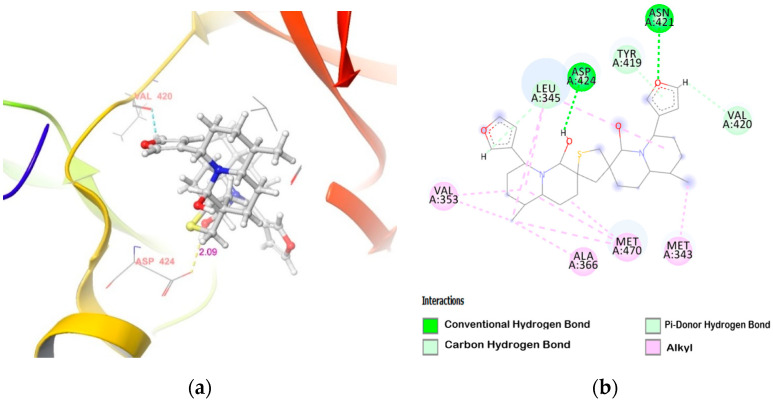
DTBN at PKCα binding pocket (**a**) 3D image and (**b**) 2D image.

DTBN binds deep into the ATP binding cleft of PKCα, surrounded by β-sheets on its sides (Figure 3), with binding score of −40.83 Kcal/mol (Table 2). At the active site, DTBN is stabilized by two hydrogen bonds and π_···_π interactions (Table 3). On the other hand, co-crystallized inhibitor NVP-AEB071 at the PKCα active site (IC_50_: 2.09 nM) is stabilized by four hydrogen bond interactions [32]. These strong interactions play a crucial role in the activity, as well as a binding affinity (−71.92 Kcal/mol).

**Figure 4:**
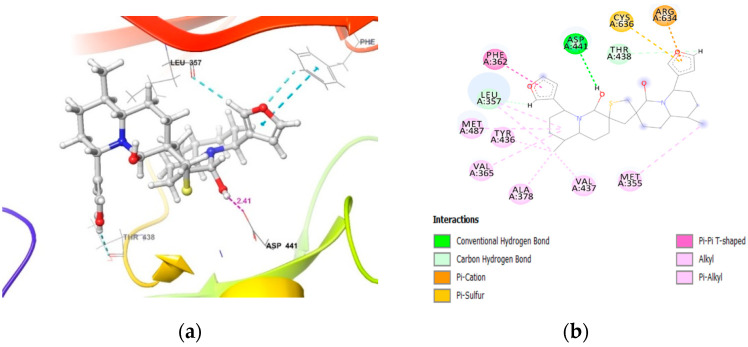
DTBN at PKCγ binding pocket (**a**) 3D image and (**b**) 2D image.

Unlike PKCα, the binding pocket of PKCγ is closed by a random coil and there is a slight shift in the β-sheets in comparison to PKCα, which resulted in fewer interactions contributing to the decreased binding score (−30.74 Kcal/mol, Table 2). At the binding site, a steric clash is observed between the furan ring of DTBN and the amino group of Arg634 in the random coil, which, in addition, contributes to the decreased binding score (Figure 4). However, the DTBN is stabilized by H-bond interaction and π-interactions, such as π_···_π and π_···_S interactions at the binding site (Table 3).

**Figure 5:**
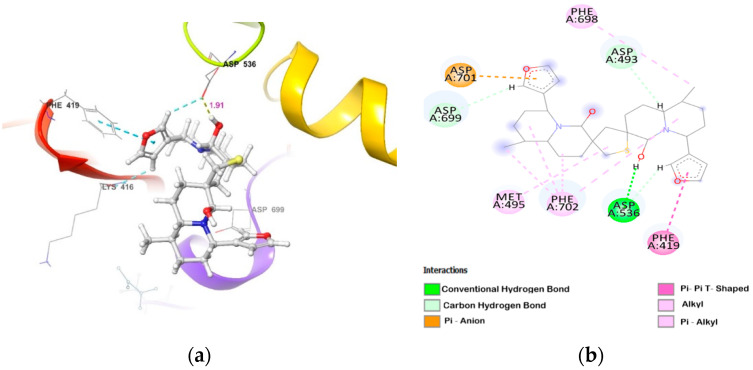
DTBN at PKCε binding pocket (**a**) 3D image and (**b**) 2D image.

**Figure 6:**
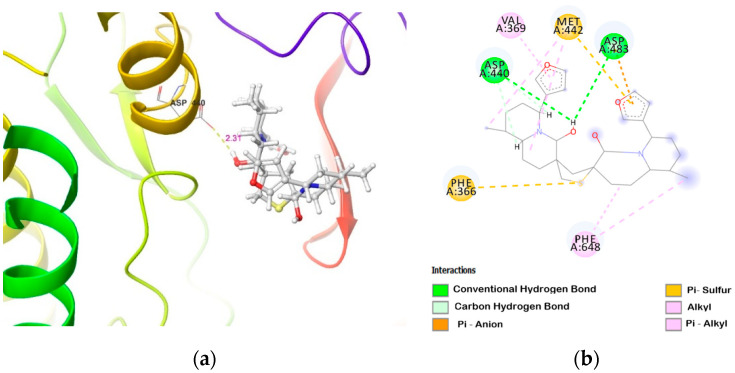
DTBN at PKCη binding pocket (**a**) 3D image and (**b**) 2D image.

**Figure 7:**
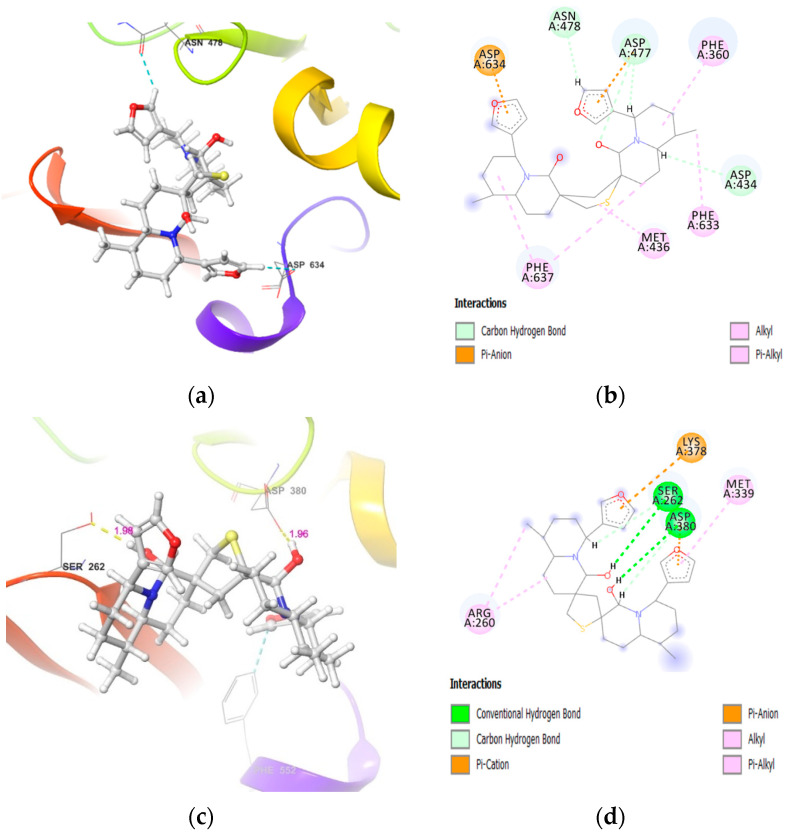
DTBN at PKCδ (top) and PKCζ (bottom) binding pockets (**a**) and (**c**) 3D images, and (**b**,**d**) 2D images.

**Figure 8:**
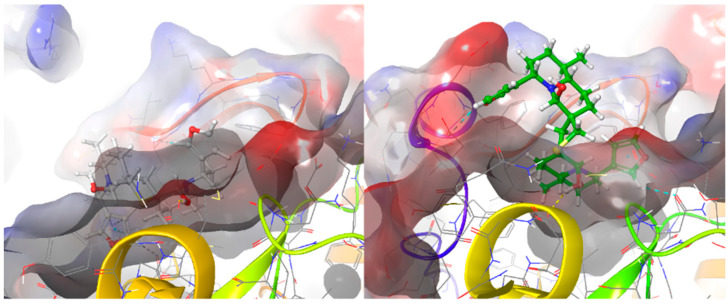
Comparison of DTBN in the binding pocket of PKCα (**left**) and PKCδ (**right**).

In correlation with in vitro results, DTBN showed a weak binding affinity with the novel and atypical PKCs (Table 2). The weak binding score was attributed to a smaller binding pocket and, hence, limited space for access and accommodation of DTBN at the active site of these kinases (Figure 5, Figure 6, Figure 7, Figure 8). Due to the smaller binding pocket, DTBN at PKCε experiences two steric clashes—one between the methyl group of DTBN and amino group (–NH) of Asp699 residue, and the other between the methylene (–CH_2_) of octahydro-1*H*-quinolizine ring in DTBN and carboxylic –OH of Asp493 residue. This weakens the ligand–enzyme interaction and contributes to a reduced binding score (−23.25 Kcal/mol, Table 2). Although it is destabilized by steric clash, it is stabilized by O–H···O and C–H···O hydrogen bonds at the active site (Figure 5).

Similarly, a smaller binding pocket of PKCη to accommodate DTBN leads to a steric clash between the octahydro-1*H*-quinolizine ring methylene (–CH_2_) group of DTBN and the amino group (–NH) of Asp645 present in the loop next to the binding pocket (Figure 6). A smaller binding pocket and steric interaction weakens the ligand–enzyme interaction and contributes to a lower binding score (−20.73 Kcal/mol, Table 2). At the active site, DTBN is stabilized by hydrogen bond and π_···_S interactions (Table 3). In comparison, the co-crystallized inhibitor naphthyridine at PKCη (IC_50_: 9 nM) is stabilized by three hydrogen bonds [33], whereas DTBN is stabilized by only one hydrogen bond. This accounts for the low binding score of DTBN.

The low binding score of PKCη in comparison to PKCε is attributed to the presence of a single-turn α-helix near the PKCε binding pocket, which results in several weak interactions with DTBN, thus providing additional stabilization for PKCε as compared to PKCη.

Alike PKCε and PKCη the binding pocket of PKCδ and PKCζ are also smaller to accommodate DTBN in the ATP binding pocket without steric clash (Figure 7), leading to fewer stabilizing interactions at the active site (−19.0 and −19.61 Kcal/mol respectively, Table 2). At the PKCζ active site, a steric clash is observed between thiophene sulfur of DTBN and the protonated guanidine amino group (–NH) of Lys378 (Figure 7 (bottom)). This contributes to its lowest binding score. However, DTBN is stabilized by two O–H···O and a C–H···O hydrogen bonds (Table 3).

In Figure 8, a comparison of DTBN at the ATP binding pocket of PKCα and PKCδ is shown. It is apparent that DTBN binds deep into the ATP binding pocket of PKCα, whereas the accommodation of DTBN at the ATP bind site of PKCδ is restricted due to a smaller binding pocket.

## 4. Materials and Methods

### 4.1. Materials

6,6′-dihydroxythiobinupharidine (DTBN) was purified from *Nuphar lutea* extracts and purchased from Sigma (St. Louis, MO, USA)/Merck (Darmstadt, Germany), cat. SMB00609. DTBN was dissolved in DMSO.

### 4.2. Methodology

#### 4.2.1. In Vitro Kinase Assay

PKC kinase assays were performed, as described [34], using myelin-basic protein (5 μg/100 μL) (MBP, #M1891, Sigma-Aldrich, St. Louis, MO, USA) as a substrate. Whole-cell lysates (75 μg per sample) of HEK-293T cells, each overexpressing different HA-tagged PKCs, were subjected to immunoprecipitation using protein A/G-agarose bead-immobilized anti-HA mAbs (#MMS-101R, BioLegend, San Diego, CA, USA). The immunoprecipitates were washed four times with Triton X-100-containing lysis buffer, and lastly with PKC kinase assay buffer (20 mM HEPES, pH 7.5, 10 mM MgCl_2,_ and 0.1 mM EGTA). The immunoprecipitates were then equally divided to Eppendorf safe-lock tubes, followed by the addition of indicated concentrations of DTBN (μg/mL) and subsequent incubation in a kinase reaction mixture (10 mM ΜgCl_2_, 20 mM HEPES, 0.1 mM EGTA, 50 μg/mL phosphatidylserine, and 5μCi γ-32P-ATP (PerkinElmer, Waltham, MA, USA), also including 100 μM cold ATP and 1.5 mM CaCl_2_ (for conventional PKCs), and incubated for 30 min at 32 °C with gentle shaking. Reactions were terminated by the addition of 5× sample buffer and boiling for 5 min, followed by SDS-PAGE (10% acrylamide gels) under reducing conditions. Samples were then transferred to nitrocellulose membranes (Sigma-Aldrich) that were developed by immunoblotting and autoradiography. After the detection of the phosphorylated substrate (^32^P-MBP) by autoradiography, the membranes were probed with anti-HA and anti-MBP mAbs (#SMI-99P, BioLegend, San Diego, CA, USA), (loading controls). ^32^P-MBP and MBP protein bands were quantified using Image Lab software (5.2.1).

#### 4.2.2. Molecular Docking and MM-GBSA Refinement

Molecular docking studies were performed using Schrödinger Maestro Suite 2020-3 (Schrödinger, LLC, New York, NY, USA). DTBN was docked to the ATP binding site of PKC isoforms. X-ray crystallographic structures of PKCα (PDB ID: 3IW4) and PKCη (PDB ID: 3TXO) are available in RCSB PDB. The structures of other PKC isoforms, δ, γ, ε, and ζ were generated using a homology modelling approach using a Swiss model online server (https://swissmodel.expasy.org/) (accessed on 20 December 2020) [35]. The homology-based models were further validated using the Swiss model inbuilt structure assessment, ERRAT, and ProSA [36,37].

#### 4.2.3. Homology Modelling and Structural Validation

**Figure 9:**
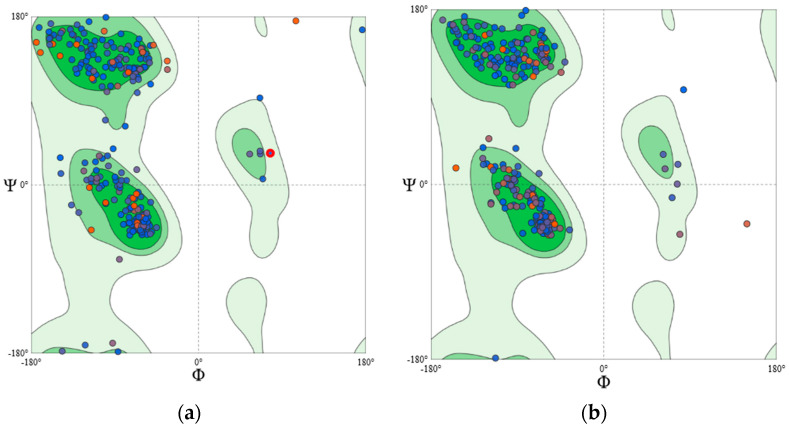
Ramachandran plot of (**a**) PKCγ and (**b**) PKC**ε**.

**Figure 10:**
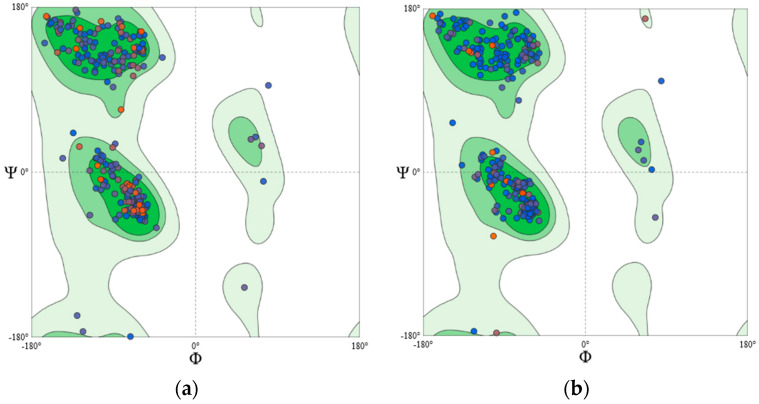
Ramachandran plot of (**a**) PKCδ and (**b**) PKCζ.

Since the crystal structures of PKC isoform δ, γ, ε, and ζ were not reported earlier, the homology modelling approach was adopted to develop their structures. The templates chosen for model generation are given in Table 4. The Q_mean_ scores of the generated models were above −2.0. Q_mean_ score provides the “degree of nativeness” of the model [38]. A Q_mean_ score near ‘0′ represents a good agreement between the predicted model structure and experimental structures of similar size. It also helps in the estimation of the quality of the generated models. Structure assessment details are given in Table 5. Further, an assessment of the Ramachandran plot (Figure 9 and Figure 10) of all the generated structures shows greater than 93% of core residues were in the favoured region; thus, suggesting generated models of PKC-isoforms were of good quality and reliable.

All the 3D X-ray crystallographic and homology modelled structures were optimized before docking using Schrödinger in-built protein preparation wizard module. Structural inconsistencies, such as missing hydrogen or incorrect bond orders, were rectified during this process. PKCα and PKCη both had co-crystallized ligand, which was chosen as the centre of the receptor grid for docking. However, in the case of homology-based models, active sites were predicted using SiteMap. Active sites with binding scores < 0.9 were rejected. The internal grid size of x × y × z was fixed as 15 × 15 × 15 Å^3^. Further, mm-GBSA refinements were carried out on the docked poses of each isoform, with a flexible residue distance of 5.0 Å.

A sitemap module was used to identify the potential binding pockets. Sitemap score is based on the potential hydrogen bonding, hydrophobicity, and pocket volume. A site score greater than 0.9 is considered as a cut-off to distinguish between the potential drug binding site and non-drug binding sites [39]. The site-score and predicted residues involved in the ATP binding pocket of PKC isoforms are given in Table 6.

## 5. Conclusions

Taken together, the present in vitro results reveal that DTBN inhibits the kinase activity of PKCs. Classical PKCs, PKCα and PKCγ, were significantly the main targets for DTBN inhibition. Molecular docking analysis of the interaction of DTBN with PKCs active sites further corroborated our results. These data point to an affinity-based effect of DTBN on PKC isozymes. The key factor to be noted here is the drug’s varying behavior to mediate stabilizing interactions at the active site of individual PKCs. In silico analysis revealed a higher binding score of DTBN upon docking with PKCα and PKCγ. This score was reduced in the cases of PKCε and PKCη, and was further reduced in the cases of PKCδ and PKCζ. The selectivity of DTBN towards PKCs could guide us in targeting pathological conditions impaired in classical PKC signaling, including cancers.

## Figures and Tables

**Table 1:** Inhibition of PKC isoforms by DTBN.

PKC Isoforms	IC_50_ Values (μg/mL)	IC_50_ Values (μM)
PKCα	0.092	0.174
PKCγ	0.089	0.168
PKCε	7.5	14.23
PKCη	>10	>19
PKCζ	9.8	18.6
PKCδ	>10	>19

**Table 2:** Results of molecular docking.

PKC Isoforms	ΔG Bind * (Kcal/mol) of DTBN	Control Molecule	ΔG Bind * (Kcal/mol)
PKCα	−40.83	NVP-AEB071	−71.92
PKCγ	−30.74	GO6983	−57.18
PKCε	−23.25	NVP-AEB071	−44.22
PKCη	−20.73	2,6-Naphthyridine	−56.52
PKCζ	−19.61	-	-
PKCδ	−19.0	NVP-AEB071	−61.35

* post mm-GBSA refinement.

**Table 3:** Interaction of DTBN in binding pocket of different PKC isoforms.

PKC Isoforms	H-Bond Interaction	π···π and π···S Interaction
**PKCα**	(i) −NH hydrogen of Asn421 and furan ring −O of DTBN (N−H···O, 2.91 Å) (ii) carboxylic oxygen (−OH) of Asp424 and hydrogen of −OH group proximal to thiophene sulphur in DTBN (O−H···O, 2.09 Å) (iii) −CH hydrogen of furan ring in DTBN and carboxylic oxygen (C=O) of Leu345 (C−H···O, 2.43 Å)	π···π interaction between phenyl and furan ring of Tyr419 and DTBN, respectively (2.24 Å)
**PKCγ**	carboxylic oxygen (−OH) of Asp441 and hydrogen of −OH proximal to the thiophene sulfur in DTBN (O−H···O, 2.41 Å)	π···π interaction between phenyl and furan ring of Phe362 and DTBN (4.80 Å). π···S interaction between S and furan ring of Cys636 and DTBN, respectively (4.29 Å).
**PKCε**	(i) side-chain carboxylic oxygen (C=O) of Asp536 and hydrogen of −OH group proximal to thiophene sulphur in DTBN (O−H···O, 1.91 Å) (ii) C−H···O aromatic hydrogen bond between (a) furan ring hydrogen of DTBN and side-chain carboxylic oxygen (C=O) of Asp536 (2.68 Å) and carboxylic oxygen (C=O) of Lys416 (2.79 Å) (b) furan ring hydrogen of DTBN and side-chain carboxylic oxygen (C=O) of amino acid residue Asp699 (2.51 Å)	π···π interaction between phenyl and furan ring of Phe419 and DTBN, respectively (4.90 Å).
**PKCη**	carboxylic oxygen (−OH) of Asp440 and carboxylic oxygen (C=O) of Asp483 involved in hydrogen bond with −OH group proximal to thiophene sulphur in DTBN (two O−H···H, 2.31 and 2.88 Å, respectively)	(i) π···S interaction between phenyl and thiophene sulphur of Phe366 and DTBN, respectively (5.02 Å). (ii) π···S interaction between furan ring and sulphur of DTBN and Met442, respectively (4.72 Å).
**PKCζ**	(i) side-chain hydroxy oxygen of Ser262 and carboxylic oxygen (−OH) of Asp380 involved in hydrogen bond with −OH groups of DTBN (two O−H···O, 1.98 and 1.96 Å, respectively) (ii) phenyl hydrogen of amino acid residue Phe552 and furan oxygen of nupharidine (aromatic C−H···O, 3.23 Å)	-
**PKCδ**	-	-

**Table 4:** Homology models properties.

Protein Model	Template	Seq. Identity	Seq. Similarity	Coverage	GQME	Q_mean_	Range
**PKCγ**	3IW4.1.A	76.99%	0.55	0.49	0.35	−1.78	345–686
**PKCδ**	5f9e.1.A	72.99%	0.53	0.49	0.32	−0.41	343–674
**PKCε**	3txo.1.A	72.02%	0.53	0.46	0.32	−0.32	406–733
**PKCζ**	5li1.1.A	84.15%	0.57	0.59	0.39	−0.63	246–585

**Table 5:** Homology models structure assessment.

Protein Model	MolProbity Score	Ramachandran Favoured	Ramachandran Outliner	ERRAT Overall Quality Factor	ProSA Z-Score
**PKCγ**	1.94	93.53%	0.59%	93.91	−8.0
**PKCδ**	1.08	96.97%	0.00%	89.35	−8.59
**PKCε**	1.31	96.32%	0.61%	88.56	−8.06
**PKCζ**	0.87	97.93%	0.30%	92.76	−9.73

**Table 6:** The site-score and residues involved in the ATP Binding Pocket of PKC isoforms.

PKC Isoforms	Site Score	Predicted Site Amino Acid Residue Numbers
**PKCγ**	1.042	357,358,361,362,365,378,380,399,403,418,434,435,436,437,438,439,440,444,445,448,482,484,485,487,490,497,498,499,631,633,634,635,636,638, 639
**PKCε**	1.078	414,415,418,419,422,435,437,456,460,470,486,487,488,489,492,493,495,496,499,532,534,536,537,539,549,550,551,552,553,697,698,699,702
**PKCζ**	1.044	258,259,260,261,262,263,264,266,279,281,314,330,331,332,333,337,339,340,343,376,378,380,381,383,393,394,396,397,414,548,549,551
**PKCδ**	1.037	355,356,358,359,360,361,362,363,376,378,379,380,381,384,385,388,390,392,393,394,396,397,399,401,411,422,427,428,429,430,434,436,437,440,471,472,473,475,477,478,480,490,491,492,493,494,495,496,497,498,507,633,634,637,644,645,646

## Data Availability

Not applicable.

## Sample Availability

Samples of the compounds are available from the authors.
